# Efficient Extraction and Recovery of Astaxanthin from Bacterial Sources Using Fatty Acids

**DOI:** 10.1021/acsomega.6c03557

**Published:** 2026-10-29

**Authors:** Vittoria Sapone, Mina Soleimani Hassanabadi, Eryka Mrotek, Marco Bravi

**Affiliations:** Department of Chemical Engineering Materials Environment, 9311 Sapienza University of Rome, via Eudossiana 18, Rome 00184, Italy

## Abstract

Natural astaxanthin is heavily preferred over synthetic alternatives for both food and pharmaceutical applications due to its superior bioactivity. However, conventional recovery methods rely on toxic volatile organic solvents (VOCs) and energy-intensive thermal distillation. This study presents an integrated, nonthermal biorefining strategy for *Paracoccus carotinifaciens* using short- and medium-chain food-grade fatty acids. The proposed “one-pot” process utilizes the pH-controlled dissociation of these solvents to simultaneously extract and recover the ketocarotenoids in a single vessel. Propanoic acid achieved the highest relative initial extraction efficiency (∼100%), outperforming acetic (88%) and butanoic (83%) acids. Subsequent alkaline phase-shifting triggered a thermodynamic “dissolution-to-precipitation” switch, yielding quantitative recovery (>98%) of carotenoids from propanoic, butanoic, and hexanoic acid solutions. High-performance liquid chromatography suggested the preliminary structural preservation of a highly enriched fraction of astaxanthin, adonirubin, and canthaxanthin relative to the starting bacterial biomass. Furthermore, a conceptual proof of principle of solvent recovery was demonstrated with hexanoic acid, achieving 80% bulk solvent recovery strictly via phase separation. This methodology completely eliminates VOCs and inherently bypasses thermal degradation, offering a highly sustainable, scalable route for industrial microbial pigment production.

## Graphical abstract

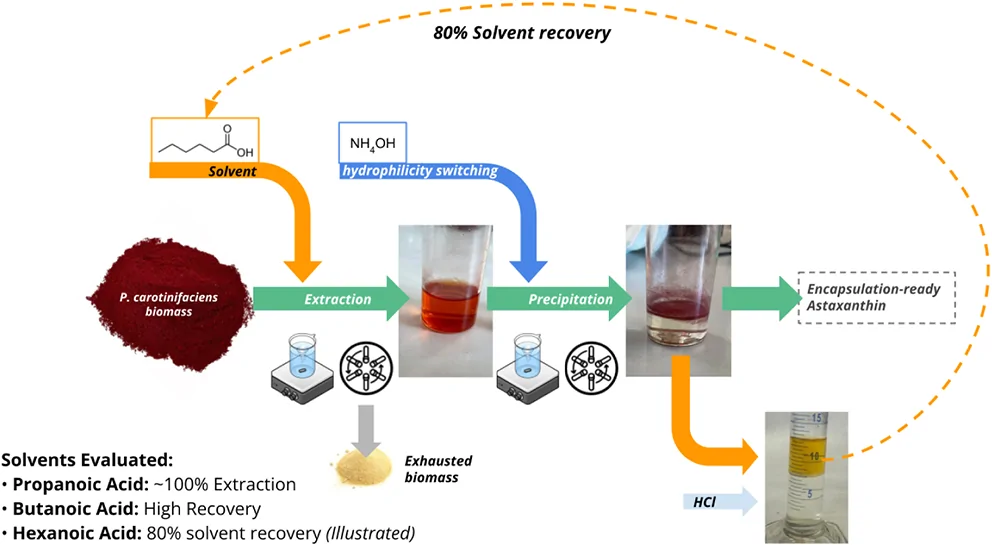

## Introduction

Astaxanthin (3,3′-dihydroxy-β,β-carotene-4,4′-dione) is a high-value ketocarotenoid widely utilized in the food, cosmetic, and pharmaceutical industries due to its exceptional antioxidant properties.^1–3^ While synthetic astaxanthin currently dominates the global market, increasing consumer demand for natural products has driven the commercial exploitation of microbial sources, notably the aerobic bacterium *Paracoccus*.^4,5^

The industrial recovery of intracellular carotenoids has traditionally been bottlenecked by the downstream processing stage. Conventional extraction methods rely heavily on volatile organic solvents, which pose significant environmental, health, and safety risks while simultaneously requiring energy-intensive thermal distillation for product isolation and solvent recovery.^6,7^ Consequently, there is a critical need for process intensification strategies that align with green extraction principles by substituting hazardous volatile organic solvents with sustainable alternatives.^8–11^

To overcome the thermodynamic limitations of traditional extractions, switchable hydrophilicity solvents (SHS) have emerged as highly effective alternatives capable of reversibly altering their phase behavior upon a chemical trigger. We recently validated this concept using amine-based SHS for microbial astaxanthin recovery (Sapone et al., 2023); however, the inherent toxicity of synthetic amines limits their downstream application in food and pharmaceutical formulations. Building upon this conceptual framework, naturally occurring short- and medium-chain fatty acids present an unexploited, food-grade alternative. While medium-chain fatty acids (e.g., hexanoic acid) retain classic SHS liquid–liquid biphasic behavior, the highly water-miscible shorter-chain homologues (C2–C4) operate via a more fundamental thermodynamic shift. For these solvents, the core concept is the pH-controlled dissociation of the carboxylic acid. Applying an alkaline trigger converts the protonated fatty acid into a highly polar carboxylate salt, inducing a massive spike in ionic strength and bulk polarity. This “salting-out” effect drastically reduces the solvating power of the medium, enabling a clean “dissolution–precipitation” switch. By forcing the antisolvent precipitation of the pigment through pH adjustment rather than heat, the thermal degradation of astaxanthin is inherently bypassed.

## Materials and Methods

### Biological Materials

Dried biomass of *Paracoccus carotinifaciens* (Panaferd AX) was obtained commercially. The industrially produced *Paracoccus carotinifaciens* biomass utilized in this study was supplied in a dried, powdered form. To accurately evaluate the direct permeation capability of the selected fatty acids, the biomass was used as received, without prior mechanical disruption, milling, or cell-wall pretreatment. *P. carotinifaciens* is a Gram-negative bacterium characterized by a high protein (∼52.5%) and carotenoid (∼4.1%) content. Approximately 50% of the total carotenoid fraction corresponds to astaxanthin.^12^

### Solvents

Analytical-grade linear saturated fatty acids were used as solvents: acetic (C2), propanoic (C3), butanoic (C4), and hexanoic (C6) acids. Additionally, the branched isomer 2-ethylhexanoic acid (EHA) was tested. All fatty acids were purchased from Alfa Aesar (USA). Alkaline solutions were prepared using analytical grade NaOH (Carlo Erba, Italy) and aqueous NH_4_OH (28% w/w, Honeywell Fluka, USA). Acetone (analytical standard) was purchased from Sigma-Aldrich (USA). All reagents were of analytical grade and used without further purification.

### Extraction Experiments

Astaxanthin extraction followed this protocol.^13^ Biomass (0.25 g) was suspended in 7.5 mL of the respective solvent (solid-to-liquid ratio 1:30 w/v) and agitated via magnetic stirring for 3 h at 25 °C. Following extraction, phase separation was performed by centrifugation at 1165 × g for 10 min, and the supernatants (liquid extracts) were collected. For the subsequent pigment recovery phase via alkaline phase-shifting, these liquid extracts were maintained under continuous magnetic stirring at 500 rpm at room temperature (25 °C).

### Astaxanthin Recovery Protocol

Carotenoid recovery was induced by adding an alkaline aqueous solution to the fatty acid extracts to trigger deprotonation and phase separation. Two bases were evaluated:

- Ammonium hydroxide: Aqueous NH_4_OH (28% w/w, 14.8 M) was added at a molar ratio of 4:1 (base:acid), demonstrated to maximize phase separation efficiency in fatty acid–based phase separation during β-carotene extraction.^15^
- Sodium hydroxide: Aqueous NaOH (14% w/v, 3.5 M) was added at a molar ratio of 0.63:1 (base:acid) as this stoichiometric condition has been recently demonstrated to maximize phase separation efficiency in fatty acid–based switchable solvents.^16^

Following alkali addition to trigger the thermodynamic switch, the mixture was allowed to equilibrate under gentle stirring for 15 min to ensure precipitation of the target ketocarotenoids and then subjected to centrifugation at 1165 × *g* for 10 min to completely separate and isolate the enriched carotenoid pellet from the aqueous salt phase, and the isolated products were stored at 4 °C until further analysis. The supernatant was filtered and analyzed by UV–vis spectrophotometry, and the Recovery Yield (%) was calculated as the percentage decrease in the total absorbance of the liquid phase before and after precipitation. All extraction and recovery runs were performed in triplicate

### Analytical Methods

Total carotenoid concentration in the liquid extracts was quantified by UV–vis spectrophotometry (Shanghai Mapada UV-1800 PC) at λ_max_ ≈ 470 nm. Concentrations were expressed as equivalent astaxanthin using a calibration curve derived from a pure standard.

To quantify the performance of the fatty acids without performing a complete solid-phase mass balance, the baseline for maximum extractable carotenoids (representing 100% extraction efficiency) was established through an exhaustive control extraction using acetone. Acetone is widely documented in the literature as the gold-standard benchmark solvent for total carotenoid recovery from microbial matrices,^14^ including specific applications for *P. carotinifaciens*. Consequently, all extraction efficiencies and recovery yields reported for the fatty acid systems in this study are expressed as relative efficiencies compared to this exhaustive acetone baseline.

Carotenoid profiling was performed via Normal-Phase High-Performance Liquid Chromatography (HPLC). The system was equipped with an isocratic pump, column oven (25 ± 2 °C), refrigerated autosampler (5 ± 2 °C), and a variable-wavelength UV–vis detector set to 470 nm. Chromatographic separation was achieved using two silica columns connected in series (Inertsil SIL-100 Å, 5 μm, 2504.6 mm) protected by a precolumn of the same stationary phase. The mobile phase consisted of *n*-Hexane:Tetrahydrofuran:Methanol (40:20:1 v/v) delivered at a flow rate of 1.0 mL/min.

Sample preparation followed a specific protocol to ensure quantitative recovery from the biomass. *P. carotinifaciens* biomass (50–80 mg) was humidified with water (150 μL), centrifuged, and extracted with a Tetrahydrofuran:Methanol solution (20:1 v/v). The mixture was homogenized with *n*-hexane and centrifuged (3000 rpm, 10 min). The supernatant was collected and diluted with the mobile phase prior to injection (20 μL). For the chromatographic profiling of the recovered products, the isolated solid pellet was separated from the aqueous salt phase, initially collected using hexane, and subsequently dissolved in a THF:MeOH mixture prior to filtration (0.22 μm) and injection (yielding a final analytical concentration of 3.7 mg/L for the representative C3 extract shown in Figure 3B). Peaks were identified by comparing Retention Times (RT) and Relative Retention Times (RRT) with analytical standards for β-carotene, canthaxanthin, adonirubin, and astaxanthin.

## Results and Discussion

### Extraction Efficiency of Fatty Acids

The extraction performance of linear saturated fatty acids (C2, C3, C4, C6) and the branched isomer 2-Ethylhexanoic acid (EHA) is shown in Figure 1. The results indicate a distinct relationship between the alkyl chain length and carotenoid solubilization.

**Figure 1:**
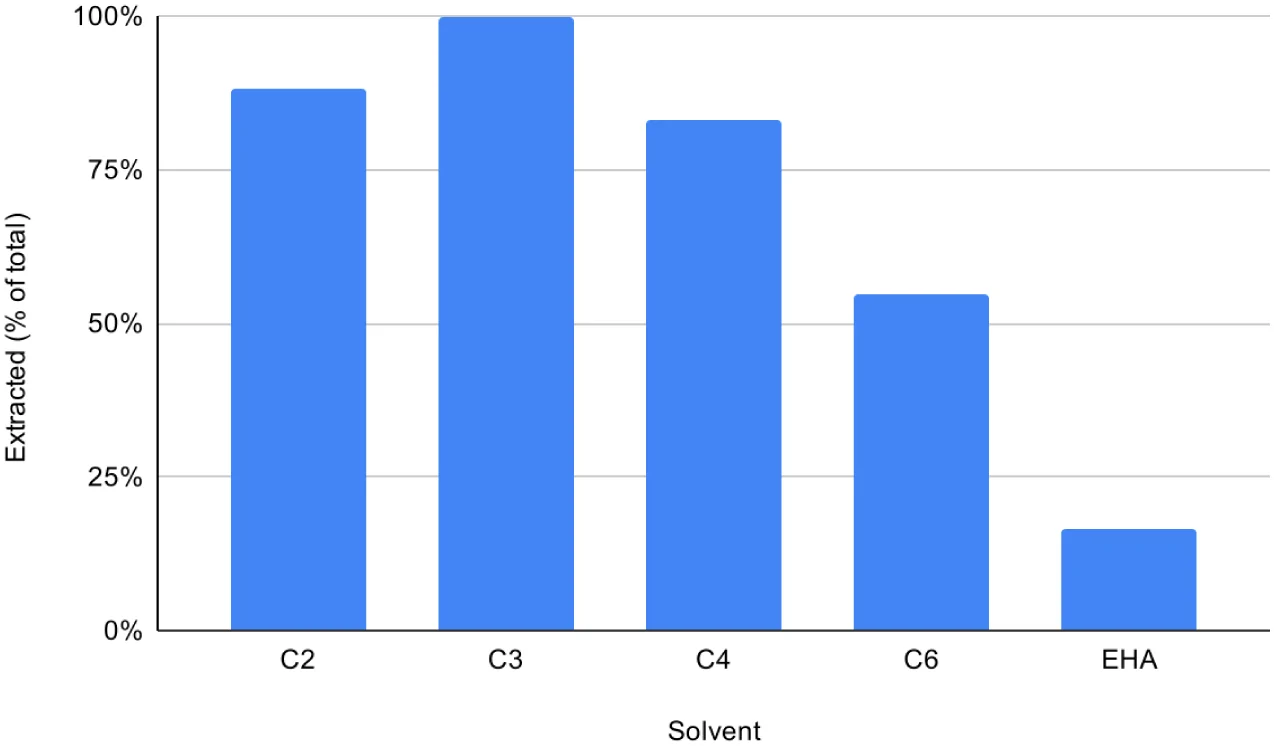
Relative extraction efficiency of intracellular ketocarotenoids from nonmechanically pretreated *Paracoccus carotinifaciens* biomass using various short- and medium-chain fatty acids. Extraction efficiencies are expressed as a percentage relative to the exhaustive benchmark extraction using acetone (established as 100%). Standard deviations for all replicates ranged from ±0.5% to ±2.8%.

Propanoic acid (C3) exhibited the highest extraction efficiency, extracting approximately 100% of the carotenoids relative to the total extractable fraction defined by the acetone control. When evaluated against the exhaustive acetone control, propanoic acid (C3) achieved the highest relative initial extraction efficiency (∼100%), significantly outperforming acetic (C2, 88%) and butanoic (C4, 83%) acids. By defining our extraction yields relative to this established industrial baseline, the data demonstrate that the direct application of propanoic acid matches the total extraction capacity of conventional volatile organic solvents. This high relative efficiency highlights the exceptional permeability of the C3 homologue into the bacterial matrix prior to the phase-shifting step. This superior performance is attributed to its intermediate polarity (Iog*P* ≈ 0.33), which provides an optimal balance for solvating the amphiphilic astaxanthin molecule while maintaining sufficient affinity for the wet biomass matrix. It is important to note that the ability of short-chain fatty acids to achieve near-quantitative extraction without upstream mechanical disruption is closely tied to the specific biological matrix. *P. carotinifaciens* is a Gram-negative bacterium, characterized by a relatively thin peptidoglycan layer that offers lower mechanical resistance to solvent permeation. Consequently, this direct “one-pot” methodology cannot be universally generalized “as is” to all carotenogenic matrices. Therefore, the exceptional direct-extraction efficiency observed here is a synergistic result of both the solvent’s penetrating power and the inherent structural vulnerability of the Gram-negative bacterial matrix.

Deviations from this optimum chain length resulted in reduced efficiency. Acetic acid (C2) achieved ∼88% extraction yield. As the chain length increased, extraction efficiency declined due to increasing hydrophobicity. Butanoic acid (C4) yielded ∼83%, while hexanoic acid (C6) reached ∼55%. This significant drop for C6 suggests that while the solvent can penetrate the biomass, its higher hydrophobicity (Iog*P* ≈ 1.9) creates a mass-transfer resistance against the aqueous environment of the cell interior, limiting the equilibrium concentration.

Finally, the branched 2-Ethylhexanoic acid (EHA) performed poorly (∼16% extraction yield). Despite having a carbon count comparable to medium-chain acids, the steric hindrance of the ethyl group likely impedes diffusion into the dense cellular matrix, effectively preventing contact with the intracellular carotenoids.

### Carotenoid Recovery via pH-Induced Precipitation

Following extraction, the recovery of the solid pigment was induced by converting the fatty acid solvent to its water-soluble salt form. This step exploits the pH-responsive nature of the carboxylic acids. The recovery yields using Ammonium Hydroxide (NH_4_OH) and Sodium Hydroxide (NaOH) are summarized in Figure 2.

**Figure 2:**
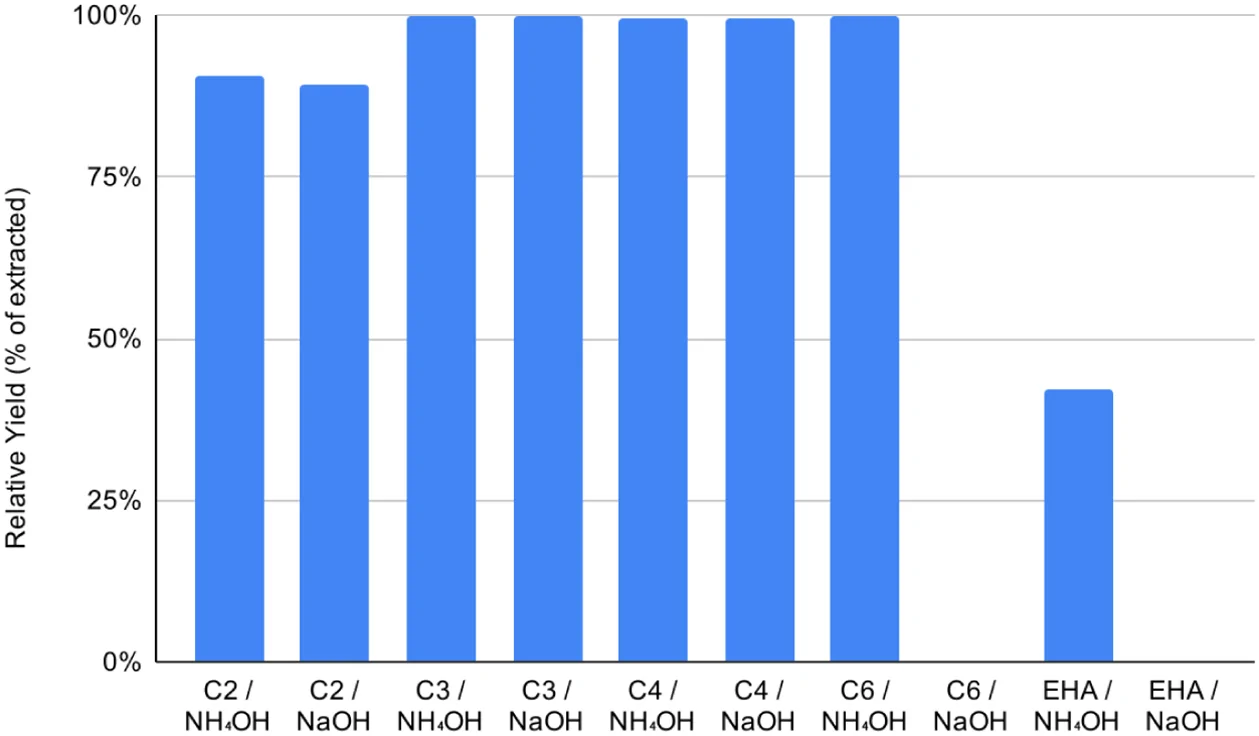
Recovery yields of ketocarotenoids following alkaline-triggered phase separation for the C3, C4, and C6 fatty acid extracts. Yields represent the mass fraction of pigment successfully precipitated and recovered relative to the total pigment present in the initial organic extract. Note the critical divergence in performance for hexanoic acid (C6) due to emulsification with NaOH. Standard deviations for all replicates ranged from ±0.3% to ±3.1%.

Two distinct mechanisms were observed depending on the fatty acid chain length: salting-out effect (C2, C3, C4) and hydrophilicity switch (C6, EHA). Acetic, propanoic, and butanoic acids are water-miscible in their neutral state. Upon base addition, the formation of high-ionic-strength carboxylate salts reduces the solubility of the hydrophobic carotenoids, forcing them to precipitate. Hexanoic acid and 2-ethylhexanoic acid are water-immiscible in their neutral state. Base addition triggers a true phase transition from a hydrophobic liquid to a hydrophilic aqueous solution (salt form) and releases the encapsulated pigment.

Propanoic acid (C3) and butanoic acid (C4) achieved near-quantitative recovery (>98%) with both bases. Visually, the supernatant became clear, indicating the complete precipitation of the pigment.

Hexanoic acid (C6) showed a critical dependence on the base used. With sodium hydroxide, the mixture formed a stable emulsion (soap formation), preventing phase separation and hindering pigment recovery. However, with ammonium hydroxide, recovery was excellent (>98%). The ammonium counterion appears to destabilize the surfactant capability of the hexanoate anion, preventing emulsification and allowing for clean precipitation.

### Product Quality and Carotenoid Profile

The HPLC chromatograms of the recovered product (from C3 extraction) versus the initial biomass are compared in Figure 3. The process selectively recovered the major ketocarotenoids, as suggested by their retention order relative to commercial standards: (1) Canthaxanthin (RT 8.46 min), (2) Adonirubin (RT 9.37 min), and (3) Astaxanthin (RT 10.36 min). Quantitatively, the chromatographic profile remained largely conserved, indicating that the recovered product constitutes a highly enriched ketocarotenoid fraction whose relative distribution mirrors the starting matrix. The relative abundance of astaxanthin in the final product was comparable to that of the fresh biomass, which strongly suggests that the mild acidic conditions of the extraction (pH 3–4) and the short alkaline exposure during precipitation did not induce extensive macrodegradation or structural rearrangement. However, these chromatographic matches represent a preliminary identification; absolute structural confirmation and the definitive exclusion of trace structural modifications or minor cis/trans isomers would require high-resolution liquid chromatography–mass spectrometry (LC-MS) in future scale-up optimizations. Minor unidentified peaks appearing at lower retention times (<5 min) are likely degradation byproducts or lipid residues, but these constitute a negligible fraction (<5%) of the total peak area (Table S2).

**Figure 3:**
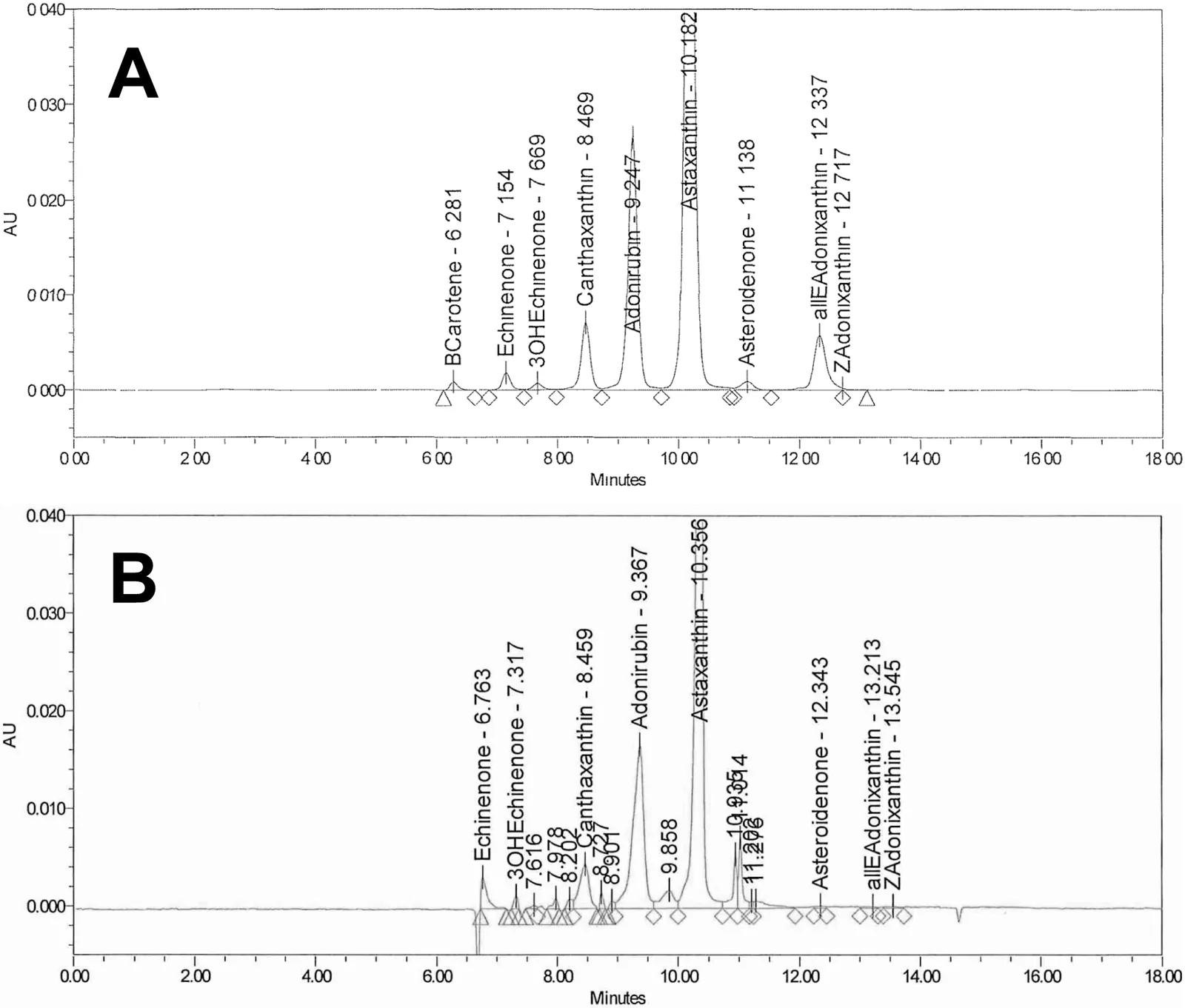
High-performance liquid chromatography (HPLC) profiles recorded at 470 nm. (A) Reference chromatogram of the complete ketocarotenoid profile extracted from the fresh *Paracoccus carotinifaciens* biomass using the exhaustive acetone baseline (total carotenoid concentration: 36.6 mg/L). (B) Chromatogram representing the highly enriched ketocarotenoid fraction recovered via alkaline precipitation from the propanoic acid (C3) extract. For this analysis, the recovered solid precipitate was isolated, initially collected with hexane, and redissolved in a THF:MeOH mixture to a concentration of 3.7 mg/L prior to injection. Numbered peaks indicate the major targeted ketocarotenoids, identified by retention time matching with commercial standards: Canthaxanthin (RT 8.46 min), Adonirubin (RT 9.37 min), and Astaxanthin (RT 10.36 min).

### Visual Process Monitoring

The macroscopic changes during the “one-pot” operation confirm the efficiency of the phase transitions. Figure 4 illustrates the sequential stages for the hexanoic acid (C6) system. Upon mixing the pale-orange biomass with the acid, the solvent immediately turns deep red (Figure 4a), indicating rapid solubilization. The addition of the alkaline trigger (NH_4_OH) causes an instantaneous color shift (Figure 4b); the deep red supernatant becomes transparent, while the pigment aggregates into a dark red precipitate. The absence of suspended turbidity in the aqueous supernatant confirms that the recovery is driven by a distinct solubility switch rather than partial dispersion.

**Figure 4:**
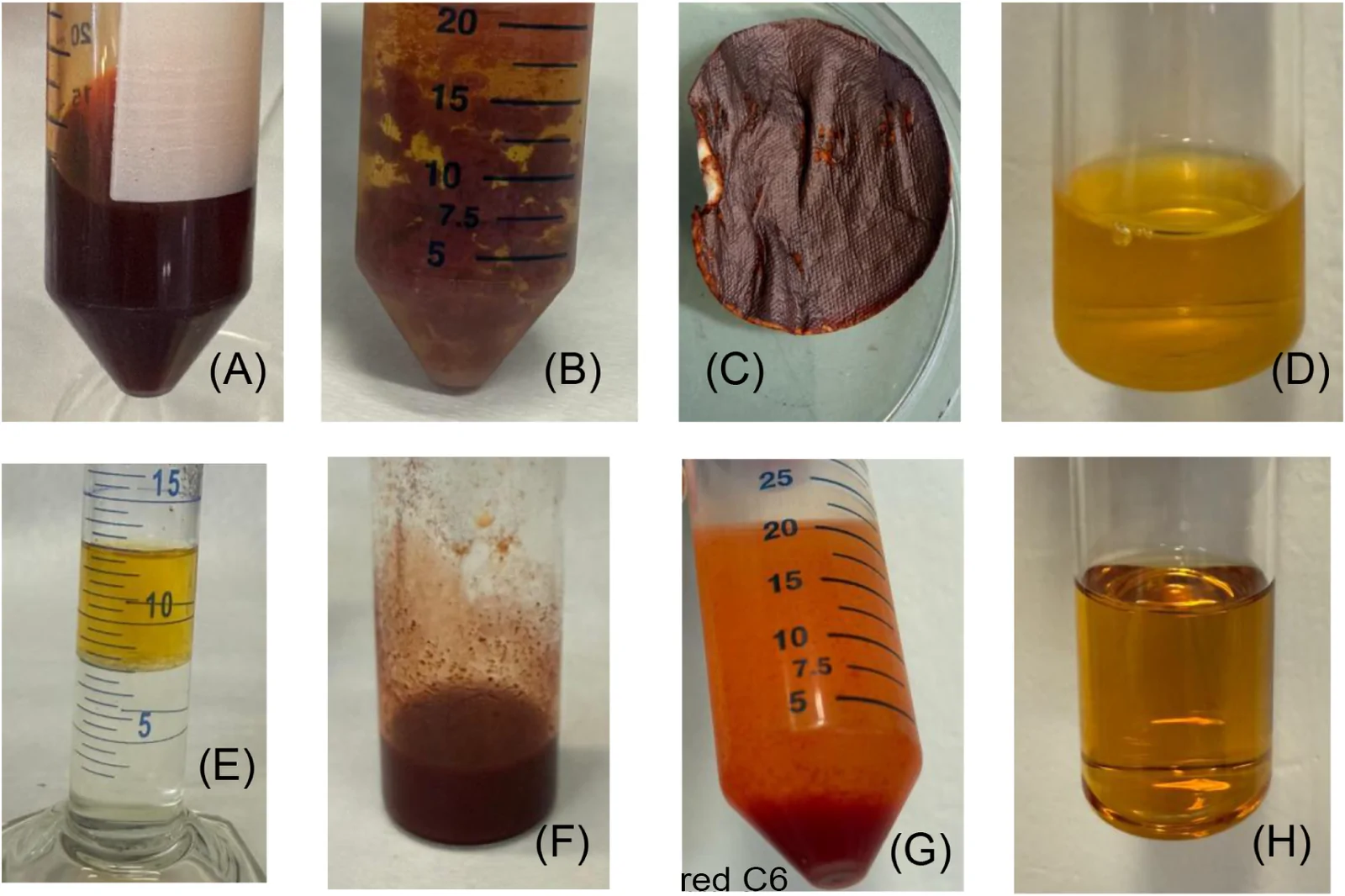
Timeline of visible results during the key steps in astaxanthin extraction and recovery with hexanoic acid, repeated over two cycles with recycled hexanoic acid. (A): Hexanoic acid-based astaxanthin extract obtained with fresh hexanoic acid (15 mL @ S/L = 1:30); (B): hexanoic acid-based astaxanthin extract precipitation after NH_4_OH conditioning; (C): solid astaxanthin separated by vacuum filtration of the suspension shown in subfigure (B); (D): supernatant from subfigure B past vacuum filtration; (E): phase splitting after back-switching of supernatant in subfigure D with HCl (the upper phase is water-saturated C6 for recycling, lower phase is ammonium hexanoate-saturated water); (F): hexanoic acid-based astaxanthin extract obtained with recycled hexanoic acid (still in contact with biomass waste; solvent volume: 3 mL @ S/L = 1:30); (G): recycled hexanoic acid-based astaxanthin extract precipitation after NH_4_OH conditioning; (H): supernatant from subfigure G past vacuum filtration. All the pictures in this figure were taken by manuscript author Marco Bravi during the experimental work.

### Engineering Implications and Solvent Selection

The experimental results highlight a critical trade-off between extraction capacity and downstream processability, which is a determining factor for industrial scale-up. Propanoic acid (C3) demonstrated the highest solvation capacity, matching the performance of acetone with a quantitative recovery. However, its complete miscibility with water implies that solvent recycling would require distillation to separate the dilute acid or break the aqueous azeotrope, which incurs significant energy costs.

In contrast, hexanoic acid (C6) offers a distinct operational advantage despite its lower single-pass yield (∼55%). As demonstrated in the subsequent recycling study, the ability to recover most of the solvent via simple phase separation eliminates the latent heat of vaporization requirement associated with distillation. In an industrial context, this reduction in thermal energy demand presents a potential operational advantage that may offset the lower extraction yield. While a rigorous techno-economic assessment is required to define the precise break-even point—similar to recent economic evaluations of other one-pot bacterial astaxanthin extractions^17^—the elimination of distillation represents a clear simplification of the process unit operations.

A further key advantage of this protocol is the substitution of thermal evaporation with a chemical potential shift via a pH adjustment. This allows for the recovery of heat-sensitive ketocarotenoids at ambient temperatures, preserving the original isomeric profile of the biomass. Consequently, the choice between C3 and C6 depends on the specific economic weighting of raw material recovery versus energy consumption in the final process design.

Within this experimental framework, the two solvents were intentionally assigned different methodological roles. Propanoic acid (C3) was utilized as the baseline for chemical profiling and product quality assessment because of its superior matrix penetration. Conversely, hexanoic acid (C6) was deployed strictly as a processing model to validate the macroscopic physical mechanics of nonthermal phase separation. Because C6 was evaluated purely to establish the thermodynamic boundary of liquid–liquid decantation, detailed HPLC profiling was restricted to the high-yielding C3 extract.

### Comparison with Alternative Extraction Methods

Considering the comparative ease of obtaining astaxanthin and carotenoids from bacterial biomass compared to competing carotenoid sources, various methods have been employed for the recovery of carotenoids from *Paracoccus carotinifaciens*, each having its own advantages and limitations. While traditional solvent extraction remains a common approach, advanced techniques like supercritical fluid extraction (SFE), ultrasound-assisted extraction (UAE), and microwave-assisted extraction (MAE), as well as the use of green solvents such as deep eutectic solvents (DES) and ionic liquids (ILs), are claimed to offer promising avenues for enhancing efficiency, reducing environmental impact, and improving the quality of the extracted carotenoids. Some published approaches and their claimed yields are reported in Table 1. The choice of the most suitable method depends on various factors, including the desired carotenoid profile, cost considerations, scalability, and environmental sustainability goals.

**Table 1:** Carotenoid Productivity Obtained from Bacterial Sources by Some Published Processes

Carotenoid(s) Recovered	Extraction Method	Solvent(s)	Conditions	Recovery Value (%)	Source Reference
Astaxanthin, Adonirubin, Adonixanthin	Supercritical CO_2_ Extraction (SC–CO_2_)	CO_2_, Ethanol (entrainer)	120 °C, 30 MPa	56.4 (Astaxanthin)	^18^
Astaxanthin-rich extract	Ultrasound-Assisted Extraction (UAE)	Choline chloride:acetic acid (DES)	65 °C, 8 min, Solid/liquid ratio 0.05 g/mL, Amplitude 15%	>95 (Total ARE)	^19^
Total Carotenoids (from G. alkanivorans)	Solvent Extraction	Tributyl(ethyl)phosphonium diethyl phosphate, Ethyl acetate	22 ± 2 °C	Near complete	^20^
Canthaxanthin, Total Carotenoids	Ultrasound-Assisted Extraction (UAE)	Ethanol (96%), Glyceryl monostearate (0.5%)	35 °C, 90 min, Solvent/material ratio 9.5:1 (v/w), Ultrasonic power 145 W	14.95 (Canthaxanthin), 18.21 (Total)	^21^
Astaxanthin, Adonirubin, Adonixanthin	Solvent Extraction	N,N-dimethyl cyclohexylamine (DMCHA)	Single-pot process	62.5 (as equivalent ASX)	^22^
Astaxanthin, Adonirubin, Adonixanthin	Microwave-Assisted Extraction (MAE) with NaDES	DL-Menthol:Acetic Acid	0.5 g/mL, Duty cycle at 40%, 120 °C (final)	32% (as equivalent ASX)	^23^

### Solvent Recovery and Process Scale-Up Considerations

To maintain a clear boundary between experimental findings and theoretical process engineering, a distinction must be drawn between the nonthermal phase recovery physically demonstrated in this study and the conceptual mass-balance closure strategies evaluated for industrial scale-up. The physical recovery of hexanoic acid (C6) via acid-triggered phase splitting (demonstrated in Figure 4) represents a laboratory-validated proof of concept. Conversely, thermal distillation of the residual aqueous phase and open-loop coproduct valorization represent conceptual scale-up frameworks intended to guide future pilot evaluations.

The proposed biorefining strategy is designed for sustainability through the circular use of fatty acids. The process involves two primary chemical transitions: the initial deprotonation of the fatty acid to trigger carotenoid precipitation, followed by the reprotonation of the resulting carboxylate salt to recover the solvent for reuse.

For the recovery and recycling of hexanoic acid (C6), two distinct pathways were considered:

1 Mineral acid route (experimentally validated): in this study, recovery was achieved by reprotonation using concentrated hydrochloric acid (37%). This restored the hydrophobic nature of the fatty acid, inducing immediate macroscopic phase separation and yielding a spontaneous 80% bulk solvent recovery. This successfully establishes a proof-of-principle that the vast majority of the solvent can be recovered strictly without a thermal input. However, the remaining 20% represents the thermodynamic solubility limit of C6 residing in the aqueous salt phase. Consequently, a loop driven purely by spontaneous phase separation remains inherently open, indicating that a raw material makeup stream would be required to maintain steady-state operations.
2 Thermal Route (Industrial Scale-up Considerations): To address the 20% systemic loss observed in the mineral acid route and achieve a true “closed loop” at an industrial scale, thermal recovery must be integrated. Rather than distilling the entire extraction solvent bulk—as is required for conventional VOCs or fully miscible short-chain homologues—an industrial facility would close the mass balance by subjecting only the residual aqueous phase to conventional distillation. Because this thermal input is targeted exclusively at a much smaller fractional volume, the overall energy footprint of the process remains fundamentally minimized. However, the distillation route requires the thermal decomposition of ammonium carboxylates (eq 1).
1 $$R-COONH_{4\;}\Delta H\to R-COOH+NH_{3}↑$$

This route, although theoretically eliminating saline waste, presents a separation challenge, since in standard laboratory evaporative setups, ammonia and fatty acid vapors tend to recombine upon cooling.^24^ Industrial implementation would therefore require reactive distillation or stripping units to physically separate the species at high temperatures. Therefore, the overall environmental benefit of the thermal route cannot yet be assessed without a full energy balance, and the mineral acid route was selected as a robust protocol for the experimental validation in this study.

### Conceptual Perspective: Open-Loop Co-Product Valorization

While the previous routes focus on strictly closing the solvent mass balance, an economically viable “open-loop” alternative exists by valorizing the resulting aqueous phase. Because the short-chain homologues evaluated (C2–C4) are naturally occurring and food-grade, triggering the precipitation with an appropriate food-grade base (e.g., sodium hydroxide or calcium hydroxide) yields an aqueous phase rich in carboxylate salts, such as sodium propionate or calcium propionate. These specific salts are widely utilized and globally marketed as highly effective commercial food preservatives and antimicrobial agents. Instead of expending chemical reagents or thermal energy to reprotonate and recover the initial fatty acid, this aqueous phase could be directly isolated or spray-dried. This strategy completely bypasses the energy requirements of solvent recycling, effectively transforming a potential saline waste stream into a marketable secondary coproduct in strict alignment with zero-waste biorefinery principles.

## Conclusions

This study successfully demonstrated a novel two-step process for recovering astaxanthin and accompanying ketocarotenoids from *Paracoccus carotinifaciens*, identifying propanoic acid (C3) as the optimal solvent for providing the highest extraction yield. Acetic (C2) and butanoic (C4) acids also proved highly effective, achieving 88% and 83% of the exhaustive acetone baseline, respectively. The subsequent recovery step was nearly quantitative for the C3 extract, demonstrating the overall viability of the process. While extraction efficiency decreased significantly with increasing chain length, the subsequent recovery step remained highly efficient (>98%) for hexanoic acid (C6) when ammonium hydroxide was used, demonstrating that solvent polarity limits extraction more than precipitation. Subsequent precipitation with sodium or ammonium hydroxide recovered the highly enriched ketocarotenoid fraction as a solid. While both alkaline solutions worked for C2, C3, and C4 extracts, only ammonium hydroxide precipitated the pigment from C6 and EHA. The observed differences are likely due to the interplay among ketocarotenoid forms, fatty acid interactions, and deprotonation/water binding during alkaline treatment. Solvent recycling via spontaneous phase splitting was demonstrated, while total mass balance closure remains a viable scale-up perspective. The future directions of this work include a quantitative techno-economic feasibility assessment of the drafted process, pilot-scale testing of the proposed technology, and investigation of its applicability to other existing, purposely produced or waste biomasses, including emerging microalgal strains and waste materials (i.e., crustacean shells).

## Supplementary Material



## Notes

UV–vis absorption spectra of the extracts (Figure S1); HPLC quantitative analysis of the starting biomass and the final recovered product (Tables S1 and S2) (PDF)
