# Supplementary material for: Efficient Extraction and Recovery of Astaxanthin from Bacterial Sources Using Fatty Acids
Source: ACS Omega. 2026 Oct 29;11(44):68225–32. doi: 10.1021/acsomega.6c03557 (PMC13757080; doi:10.1021/acsomega.6c03557)
Supplement: Supplementary file 1 [file ao6c03557_si_001.pdf]

Supplementary Material for:

Efficient Extraction and Recovery of Astaxanthin from Bacterial Sources Using Fatty Acids

Vittoria Sapone, Mina Soleimani Hassanabadi, Eryka Mrotek, Marco Bravi*

Department of Chemical Engineering Materials Environment, Sapienza University of Rome, via Eudossiana 18, Rome, 00184, Italy

***Corresponding author**

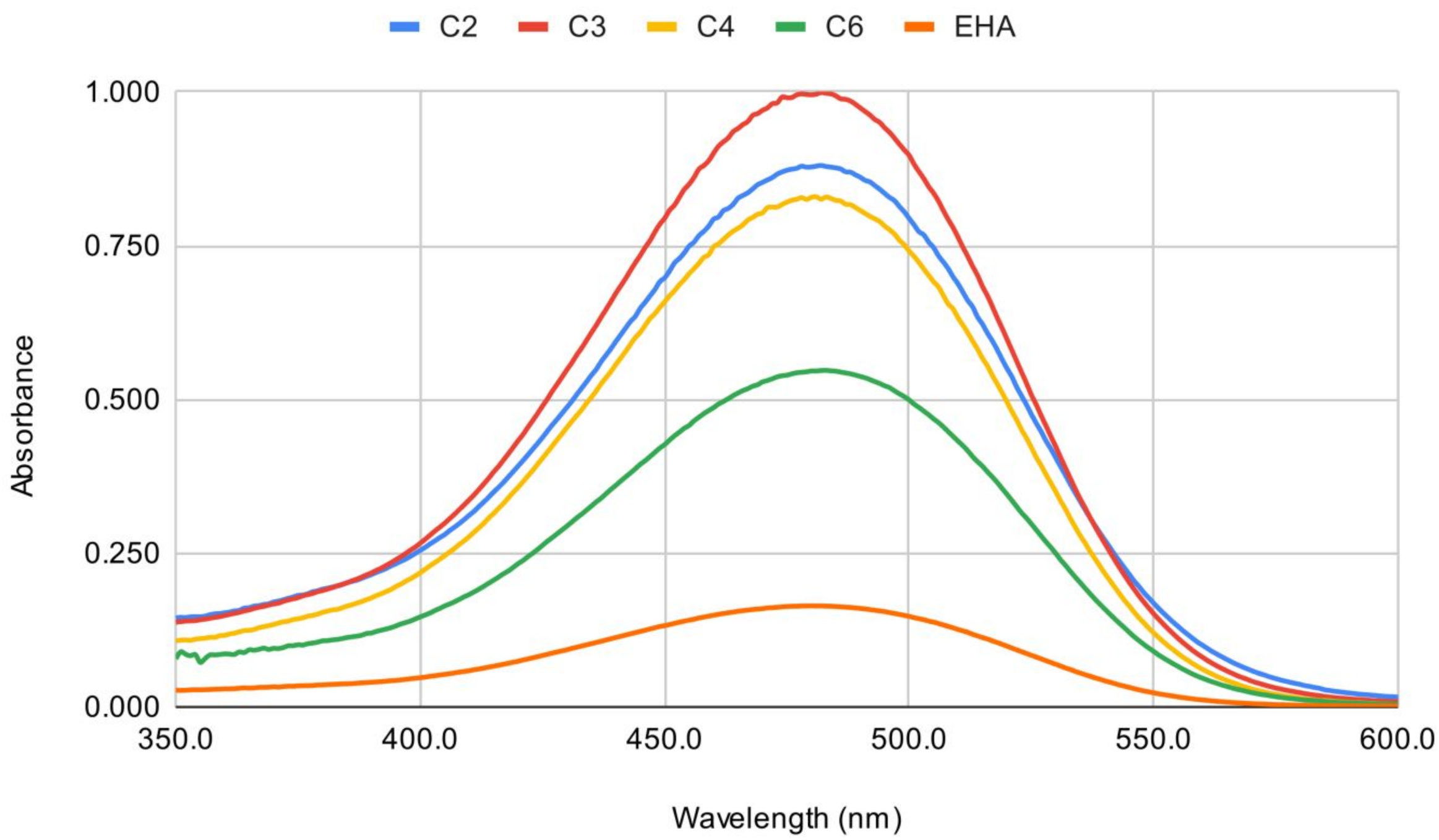

Figure S1. UV-Vis absorption spectra of the Paracoccus carotinifaciens extracts obtained using different fatty acid solvents. The spectra show the characteristic carotenoid absorption band (λ_{max} ≈ 474 nm). Note that the spectral profile remains consistent across solvents (C3, C4, C6), indicating that the solvent choice does not alter the electronic properties of the pigment.

Table S1. HPLC quantitative analysis of the starting Paracoccus carotinifaciens biomass. The total carotenoid concentration was 36.6 mg/L. Astaxanthin (RT 10.18 min) represents ~62% of the total peak area, followed by Adonirubin (22%) and Canthaxanthin (5.6%). The total

concentration of the prepared sample was 36.6 mg/L.

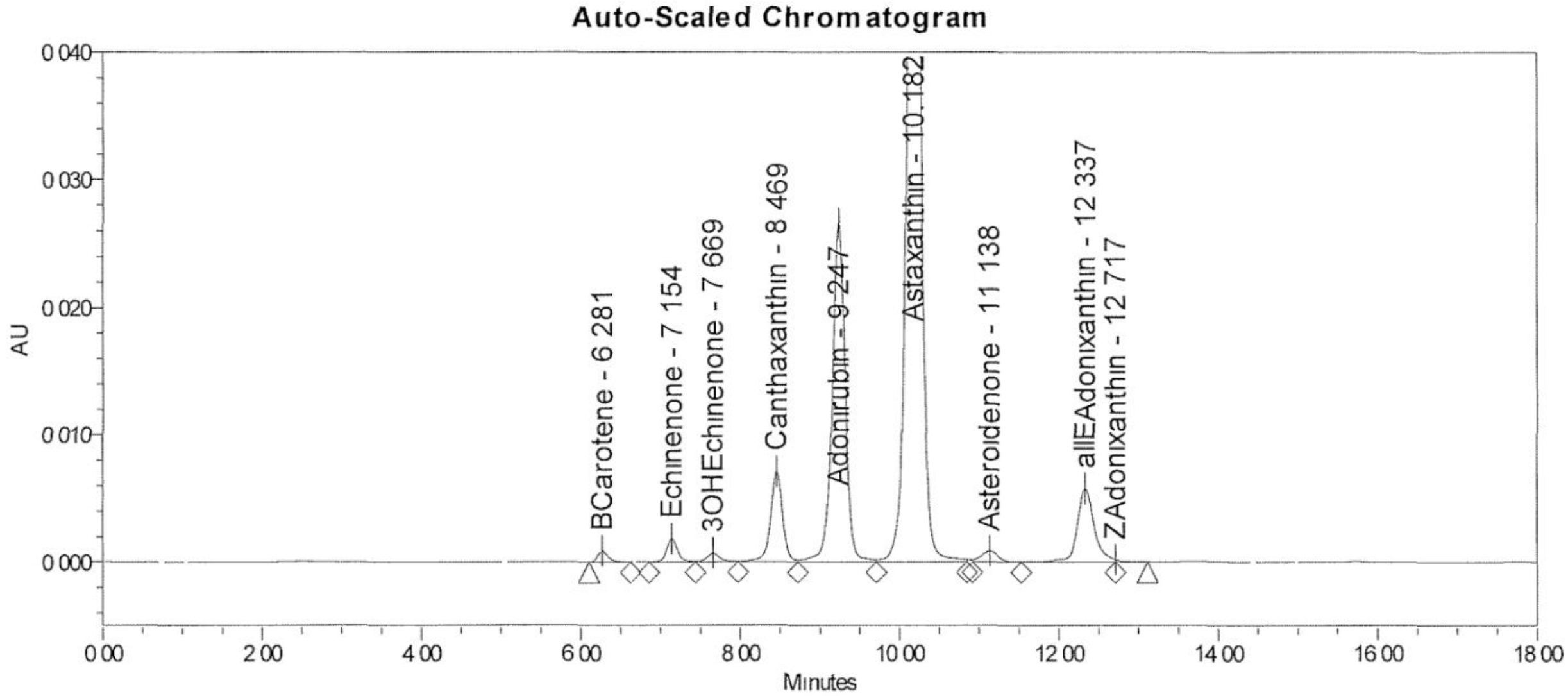

	Name	RT	RRT	Area	% Area	Amount (mg/L)
1	BCarotene	6 281	0 617	8147 2	0 60	0 2
2	Echinenone	7 154	0 703	17572 9	1 30	0 5
3	3OHEchinenone	7 669	0 753	8282 4	0 61	0 2
4	Canthaxanthin	8 469	0 832	75775 5	5 62	1 9
5	Adonirubin	9 247	0 908	301505 6	22 36	8 2
6	Astaxanthin	10 182		835534 2	61 97	22 8
7	Asteroidenone	11 138	1 094	13364 5	0 99	0 4
8	allEAdonixanthin	12 337	1 212	86750 6	6 43	2 4
9	ZAdonixanthin	12 717	1 249	1294 9	0 10	0 0
Sum						36 6

Table S2. HPLC quantitative analysis of the final recovered product. The retention times of the major peaks (Canthaxanthin ~8.5 min, Adonirubin ~9.4 min, Astaxanthin ~10.4 min) are consistent with those of the starting biomass (Table S1), strongly suggesting the preservation of the pigment profile within normal HPLC reproducibility. The total concentration of the re-suspended solid for analysis was 3.7 mg/L.

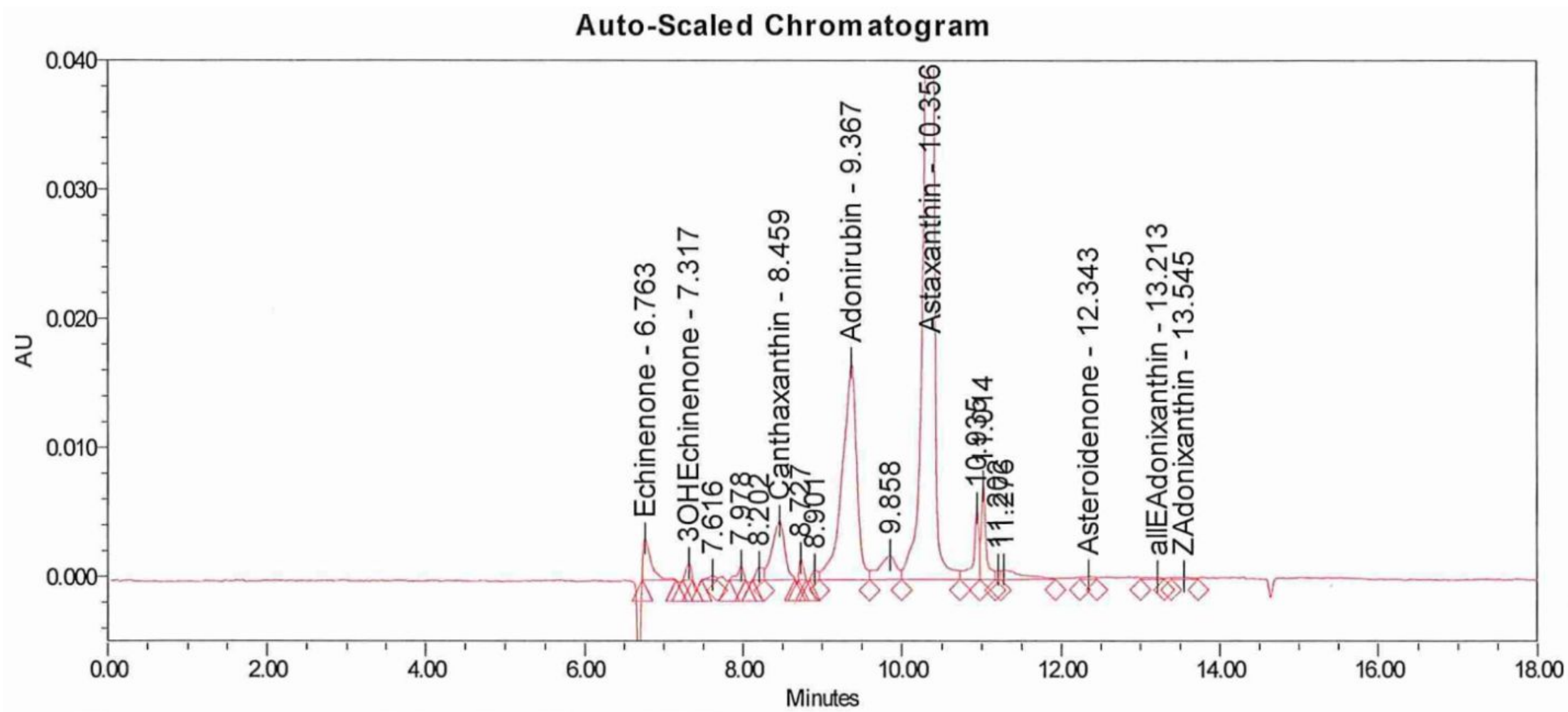

	Name	RT	RRT	Area	% Area	Amount (mg/L)
1	BCarotene	6.207				
2	Echinenone	6.763	0.653	22337.4	2.05	0.1
3	3OHEchinenone	7.317	0.707	5732.1	0.53	0.0
4	Unidentified	7.616		2232.8	0.21	
5	Unidentified	7.978		5697.0	0.52	
6	Unidentified	8.202		6055.4	0.56	
7	Canthaxanthin	8.459	0.817	50514.4	4.64	0.2
8	Unidentified	8.727		5698.1	0.52	
9	Unidentified	8.901		4031.3	0.37	
10	Adonirubin	9.367	0.905	214643.9	19.73	0.8
11		9.858		27997.4	2.57	
12	Astaxanthin	10.356		661398.8	60.80	2.6
13	Unidentified	10.935		27221.7	2.50	
14	Unidentified	11.014		30271.8	2.78	
15	Unidentified	11.202		2944.2	0.27	
16	Unidentified	11.276		14122.3	1.30	
17	Asteroidenone	12.343	1.192	2473.2	0.23	0.0
18	allEAdonixanthin	13.213	1.276	2135.6	0.20	0.0
19	ZAdonixanthin	13.545	1.308	2367.2	0.22	0.0
Sum						3.7
